# 2022 McKinney rain-on-wildfire event, dissolved oxygen sags, and a fish kill on the Klamath River, California

**DOI:** 10.1038/s41598-025-08179-9

**Published:** 2025-07-09

**Authors:** Jennifer A. Curtis, Grant S. Johnson, Josh D. Cahill, Laurel Genzoli, Cliff N. Dahm, Liam N. Schenk, John R. Oberholzer

**Affiliations:** 1 California Water Science Center, U.S. Geological Survey, Eureka, CA USA; 2 Department of Natural Resources, Karuk Tribe, Orleans, CA USA; 3 Environmental Department, Yurok Tribe, Klamath, CA USA; 4 University of Montana, Missoula, MT USA; 5 University of New Mexico, Albuquerque, NM USA; 6 Oregon Water Science Center, U.S. Geological Survey, Bend, OR USA

**Keywords:** Wildfire, Water quality, Flood wave, Sediment, Dissolved oxygen, Fish kill, Hydrology, Fire ecology, Environmental impact, Natural hazards

## Abstract

The longitudinal propagation of water-quality and ecological impairments in rivers during and after wildfires remain poorly understood. In Northern California, the 2022 McKinney Fire burned 243 km^2^ of the Klamath National Forest, with 83% of the burned area classified as moderate to high severity. During the active wildfire, a high-intensity monsoonal rain event triggered sediment-laden flooding and runoff-initiated debris flows, causing extreme water-quality impairments and a 95 km fish kill zone along the main-stem Klamath River. This rain-on-wildfire event produced a flood wave that outpaced a sediment pulse, diminishing the dilution effect of the floodwaters. A network of high-frequency water-quality sensors recorded water-quality impairments that propagated 296 km downstream. Impairments at the nearest monitoring station, situated 71 km downstream from the fire perimeter, included dissolved oxygen sags to zero (anoxia) for 5.25 h, turbidity spikes exceeding 1000 FNU, a doubling of specific conductance from 175 to 415 µS/cm (at 25 °C), and pH anomalies of 0.5 units from 7.8 to 7.3. This novel rain-on-wildfire event triggered the first flush of fire-scar material during an active wildfire, resulting in water-quality impairments unprecedented in the historical monitoring data for the river spanning 2012 to 2022. This study provides new insights into the potential role of rain-on-wildfire events in generating extreme downstream water-quality and ecological impairments in a more fire-prone future.

## Introduction

A more fire-prone future^1^ presents new challenges for managing water-quality and ecosystem services in rivers worldwide^2–5^. In the western United States (US), wildfire risks increasingly threaten water quality, making wildfire one of the most significant drivers of aquatic impairments^6–10^. The longitudinal propagation of water-quality impacts along river gradients is an understudied effect of wildfires^10^, and the mechanisms that propagate water-quality impairments in wildfire-affected rivers remain poorly understood^7,11,12^.

Although water-quality impacts of high-intensity wildfires, such as spikes in turbidity and dissolved oxygen sags to zero (anoxia), can lead to fish kills^11,13–17^, anoxia and sublethal physiological effects on fish and fish kills are among the least reported consequences of wildfire^10,18^. The scarcity of studies documenting anoxia and fish kills may reflect the scarcity of monitoring data rather than the infrequency of anoxia-related fish kills.

Post-fire longitudinal assessments of water-quality impacts are challenging because high-frequency monitoring in fire-prone areas is uncommon. Monitoring and knowledge gaps prompted recent calls for strategic and standardized monitoring to advance wildfire and water-quality science^19,20^. New strategies include high-frequency monitoring to investigate processes that drive interactions between fire-scar material and water quality, which is a key knowledge gap for predicting water-quality and ecological impairments during and following wildfires^10^.

The longitudinal propagation of wildfire-related water-quality impairments is an emerging area of research^7,12,18^. A recent study by Ball et al.^7^ used an exponential decay model and an impact threshold, defined as a decline in dissolved oxygen of > 0.5 mg/L, to estimate the total stream + river length impacted by wildfires across the western US. Between 1984 and 2014, wildfires directly affected approximately 6% of the stream + river lengths in fire-prone areas. When Ball et al.^7^ incorporated the longitudinal propagation of water-quality impacts, the estimate nearly doubled to ~ 11%, indicating that longitudinal propagation of fire-scar material elevates the risk of water-quality and ecological impairments in fire-prone ecosystems.

To the best of our knowledge, this study is the first published work documenting a rain-on-wildfire event and the longitudinal propagation of water-quality and ecological impairments. We investigate a high-intensity monsoonal rainfall event that triggered sediment-laden flooding, runoff-initiated debris flows, and the first flush of fire-scar material during an active wildfire. Subsequently, we discuss potential mechanisms for dissolved oxygen sags to zero (anoxia) and the complex interactions between the propagation of a flood wave and sediment pulse, composed of fire-scar material, which intensified downstream water-quality impairments and produced a 95 km fish kill zone.

Our specific study objectives were to examine water-quality responses to the 2022 McKinney rain-on-wildfire event and to explore patterns in the longitudinal propagation of water-quality impairments using high-frequency monitoring of turbidity, conductivity, pH, dissolved oxygen, and water temperature. This study presents a new understanding of rain-on-wildfire events and their role in producing extreme water-quality and ecological impairments in downstream receiving waters.

### Study area

**Fig. 1:**
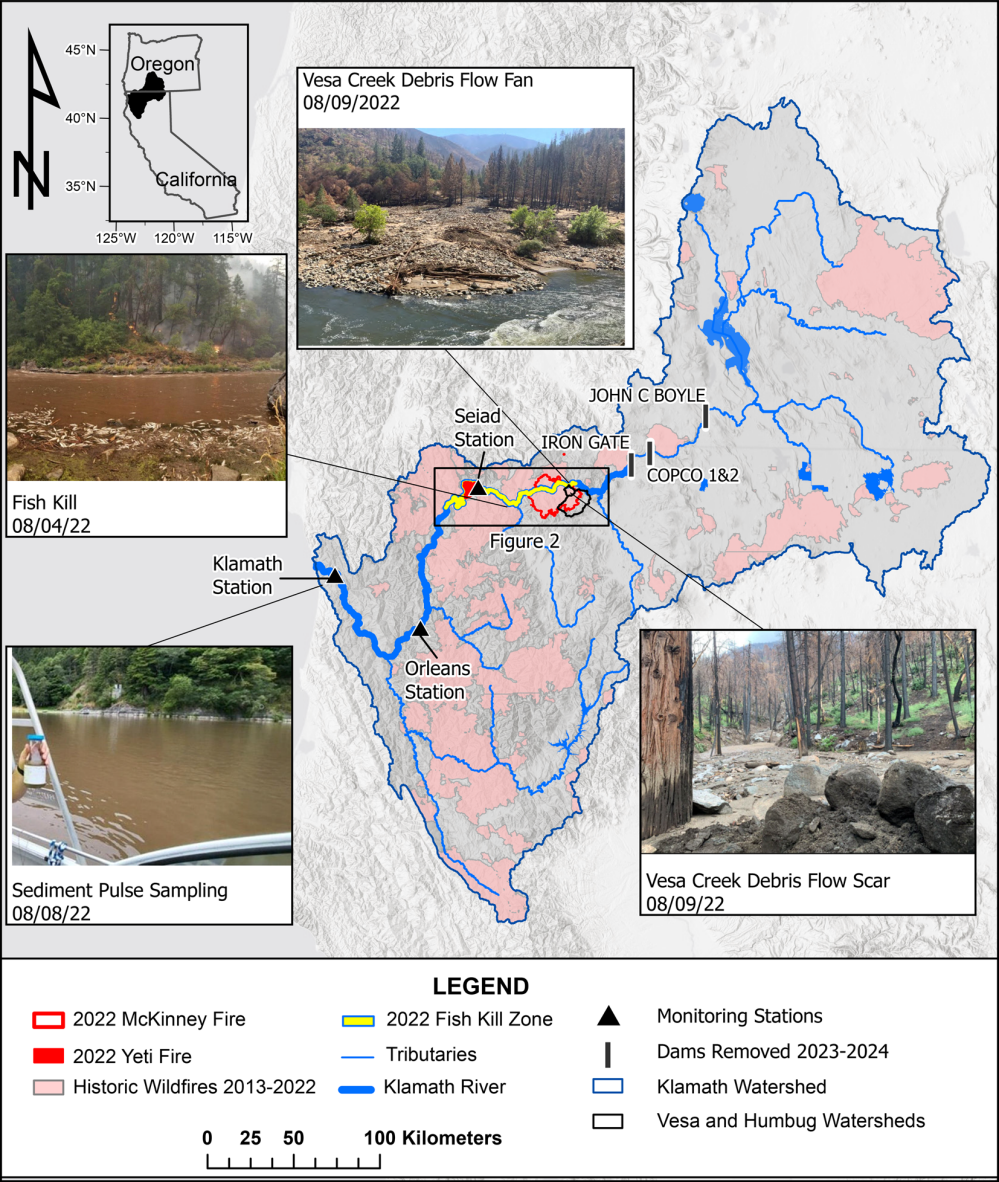
2022 McKinney rain-on-wildfire study area on the main-stem Klamath River, California. The map shows burned areas from historical wildfires between 2013 to 2022, burned areas for the 2022 McKinney Fire and 2022 Yeti Fire, major tributaries, four dams removed in 2023 and 2024, and three monitoring stations. River distances, measured upstream from the river mouth, are 282 km to the confluence of Humbug Creek, 272 km to the confluence of Vesa Creek, 211 km to the Seiad station (USGS station number 11520500), 96 km to the Orleans station (USGS station number 11523000), and 13 km to the Klamath station (USGS station number 11530500). Photos taken the week after the rain-on-wildfire event show (**a**) a post-fire debris flow (PFDF) scar in the headwaters of Vesa Creek^58^, (**b**) a debris fan at the confluence of Vesa Creek with the main-stem Klamath River^58^, (**c**) active fire and fish kill (Karuk Tribe), and (**d**) and sample of fire-scar material in transport at the Klamath station (Public Domain). Figure created using ArcGIS Pro (v. 10.6; Esri, Redlands, CA, USA).

The Klamath River drains more than 40,600 km^2^ of northern California and southern Oregon (Fig. 1). The basin is steep and rugged, the climate is Mediterranean, and precipitation mainly occurs from October to April^21^. Annual precipitation varies from 3300 mm in the west to 250 mm in the east. Seasonal snowpack typically forms above 1200 m. Most annual precipitation is produced by Pacific frontal storms during the fall and winter months. In late summer and early fall, monsoonal moisture occasionally brings short-duration thunderstorms with high-intensity rainfall.

Historically, the Klamath River was the third-largest salmon-producing river on the West Coast of the US and supported a culturally and economically significant salmon fishery^22,23^. Cumulative effects from multiple stressors have caused severe declines in native fish populations^24^. Estimated declines since the early 1900s range from 52 to 95% for endangered coho salmon (*Oncorhynchus kisutch*), over 90% for fall-run Chinook salmon (*Oncorhynchus tshawytscha*), 98% for spring-run Chinook salmon, 67% for steelhead trout (*Oncorhynchus mykiss*), and 98% for Pacific lamprey (*Entosphenus tridentatus*)^22^. Historical declines in salmon populations were attributed to fish disease^25,26^, poor water quality^27^, and insufficient scouring flows^28–30^.

Dams, reservoir operations, decades of fire suppression, and recent increases in the frequency of high-intensity wildfires played important roles in the decline of native fish populations in the Klamath River basin. In 2002, when reservoir flow releases were reduced during a dry year with a high prevalence of fish disease^31,32^, more than 33,000 adult salmon, steelhead trout, and other fish species died of disease (*Ichthyophthirius multifiliis* and *Flavobacterium columnare*). The fish kill in 2002 served as a catalyst for basin-scale water-quality monitoring. Following the 2002 fish kill, seasonal sondes were installed at existing US Geological Survey (USGS) streamflow stations. In 2018, in preparation for the removal of four hydroelectric dams (Fig. 1), turbidity sensors and suspended-sediment monitoring were added. The dam removals are expected to improve water quality and reduce fish disease^33–35^. The first dam was removed in October 2023, and the remaining three were removed during the summer of 2024.

Historic fire suppression^36^, unprecedented above-ground live (AGL) tree biomass^37^, and persistent drought^38^ increased the availability of dry fuels and the risk of high-severity wildfires throughout the Klamath River basin. Current AGL tree biomass estimates are unprecedented (250 Mg/ha) and more than double the long-term median (104–128 Mg/ha) for the past 3,000 years^37^. From 2013 to 2022, approximately 37% (7300 km^2^) of the lower Klamath River basin below Iron Gate Dam was burned by wildfires (Fig. 1). These historic wildfires include large, high-severity fires, such as the 2022 McKinney Fire, which burned 243 km^2^, as well as smaller, lower-severity fires, like the 2022 Yeti Fire, which burned 32 km^2^^43^.

During the summer of 2022, the Yeti and McKinney wildfires burned along an 84-km reach of the main-stem Klamath River (Fig. 1). The downstream Yeti Fire started on July 28, 2022, and the upstream McKinney Fire began on July 29, 2022. On August 2, 2022, a high-intensity monsoonal rainfall event occurred over the McKinney Fire, hereafter referred to as the McKinney rain-on-wildfire event.

## Methods

In this study, we used geospatial datasets to assess the timing and extent of burned areas, wildfire severity, rainfall intensity, rainfall volumes, and sediment delivery from post-fire debris flows (PFDFs). Subsequently, we utilized streamflow and water-quality observations collected at three monitoring stations (Fig. 1 and Table 1) to examine the responses of streamflow and water quality and explore patterns in the longitudinal propagation of a flood wave and sediment pulse that caused extreme water-quality and ecological impairments in the downstream receiving waters of the main-stem Klamath River.

**Table 1:** Description of three monitoring stations used to investigate patterns in the longitudinal propagation of a flood wave, sediment pulse, and water-quality impairments along the main-stem Klamath River, California.

USGS station number	USGS station name	Karuk Tribe and Yurok Tribe station name	Distance upstream from the river mouth (km)	Distance downstream from Humbug Creek (km)	Drainage area (km^2^)	Mean annual streamflow (m^3^/s)
11520500	Klamath River at Seiad Valley	Klamath River near Seiad Valley	211	71	17,980	76
11523000	Klamath River at Orleans	Klamath River near Orleans	96	186	21,950	140
11530500	Klamath River near Klamath	Klamath at Turwar	13	269	31,340	280

River distance is reported as kilometers (km) upstream from the Klamath River mouth at the Pacific Ocean^57^.

Monitoring station locations are shown in (Fig. 1).

Seiad station is located within the 95 km fish kill zone.

### Rain-on-Wildfire analysis

We used analysis-ready data (ARD) to assess the timing and extent of burned areas, wildfire severity, rainfall rates and accumulations, and sediment delivery by PFDFs during the McKinney rain-on-wildfire event. ARD is a recent concept intended to provide standardized geospatial products for analysis. We estimated the timing and extent of burned areas using the Moderate Resolution Imaging Spectroradiometer (MODIS MCD64A1) Burned Area product suite^39^. We created burn severity maps using the Normalized Burn Ratio (NBR), a remote sensing index that distinguishes healthy vegetation from burned vegetation^40^. We estimated rainfall rates and accumulations using Next Generation Weather Radar (NEXRAD) Q3 RADAR-only gridded maps^41^, and we used PFDF volumetric predictions^42^ to estimate sediment delivery from burned areas.

The MODIS-MCD64A1 product provided daily estimates of burned areas^39^. The burned area data are derived from 500 m MODIS imagery and 1 km MODIS active fire observations. This burned area product consists of polygons, each attributed with an ordinal burn day value (1–366). We downloaded the 2022 burned area polygons and merged all polygons with burn dates on or before August 1, 2022. These merged polygons represent the total extent of burned areas before August 2, 2022. The burned area maps have a spatial resolution of 500 m.

We used NBR data, with pixel values representing the difference between pre-fire and post-fire values, hereafter referred to as dNBR, to characterize fire severity for the 2022 Yeti Fire and McKinney Fire extents^43^. We classified the dNBR grids using standardized burn severity thresholds to distinguish between unburned (dNBR between −0.100 and 0.99), low severity (dNBR between 0.100 and 0.269), moderate severity (dNBR between 0.270 and 0.439), and high severity (dNBR between 0.660 and 1.30) classes^40^. The dNBR grids for the Yeti Fire and McKinney Fire have a spatial resolution of 20 m.

Because there were no nearby ground-based stations with measured rainfall for the rain-on-wildfire event, we computed rainfall rates and accumulations using NEXRAD gridded reflectivity maps. NEXRAD reflectivity-derived rainfall estimates are recorded and updated every 56 min during precipitation events. When confounding factors are well-constrained, NEXRAD estimates of rainfall rates and accumulations are commonly within 20% of nearby ground-based measurements but can differ by a factor of 2^44^.

We summarized rainfall rates and storm accumulations using NEXRAD Q3 RADAR-only gridded maps with 5–6 min estimates of rainfall rates and 1 h accumulations^41^. The 1 h accumulation grids represent the total accumulated rainfall since the last one-hour break in precipitation. We used the NOAA Weather and Climate Toolkit (WCT) App^45^ to reproject the precipitation grids from polar to planar coordinates. The reprojected rainfall grids have a spatial resolution of 120.5 m.

We used ArcPro (v.10.6) to clip and analyze the NEXRAD rainfall data and NOAA Atlas 14 to estimate the rainfall frequency^46^. The rainfall rate and 1-h accumulation grids were clipped using the burn-scar perimeters for the 2022 McKinney and Yeti fires. We used the gridded maps of 5–6 min rainfall rates to determine storm duration and rainfall intensities, and we summed the gridded maps of 1 h rainfall accumulations to compute 3 h rainfall accumulations for each pixel and estimate 3 h storm totals. We determined rainfall recurrence intervals and exceedance probabilities, using NOAA Atlas 14 searchable lookup tables.

We estimated sediment delivery from burned areas using PFDF predictions computed using standardized empirical models^52^. The volumetric predictions were derived from multiple linear regression models that estimate the likelihood and volume of PFDFs in recently burned areas^47^. These models use parameters related to the watershed shape, burn severity, soil properties, and rainfall to predict likelihood and volumetric estimates for PFDFs in response to design storms.

### Streamflow and water-quality monitoring

To explore patterns in the longitudinal propagation of water-quality and ecological impairments along the main-stem Klamath River, we used high-frequency monitoring data collected at three stations jointly operated by the USGS, the Karuk Tribe, and the Yurok Tribe (Fig. 1 and Table 1). We investigated the propagation of a flood wave using 15 min streamflow observations, and we examined the propagation of a sediment pulse composed of fire-scar material using 15 min water-quality observations. Streamflow and water-quality data were collected, archived, analyzed, and approved in accordance with standard USGS methods^48,49^. We used turbidity as a surrogate measurement for sediment^50^.

Water-quality data were collected by the Karuk Tribe and Yurok Tribe using multiparameter sondes (EXO2, YSI Inc., Yellow Springs, Ohio, USA). The sondes were equipped with a central wiper, a combined water temperature (°C) and specific conductance (μS/cm at 25 °C) sensor, an optical dissolved oxygen (mg/L) sensor, a pH sensor, and an optical turbidity sensor (FNU). The turbidity sensors have a maximum read range of 2,000 FNU; however, accuracy declines above 1,000 FNU^51^. The sondes were programmed to collect a 40 s burst of data from each sensor, which was then averaged and recorded for each 15 min timestamp. Biological activity and growth, referred to as biofouling, can interfere with sensor readings. The sondes were programmed to wipe the sensors before each measurement, and the sensors were cleaned monthly to minimize biofouling.

All streamflow and water-quality observations used in this study are publicly available (Table 1). The 15 min streamflow data were collected by the USGS and published in the National Water Information System^52^. The 15-min water-quality data at the Seiad (USGS station number 11520500) and Orleans (USGS station number 11523000) stations were collected and published by the Karuk Tribe^53^. The 15 min water-quality data at the Klamath station (USGS station number 11530500) were collected and published by the Yurok Tribe^54^. Turbidity data at all three monitoring stations, collected by the Karuk and Yurok Tribes, were furnished to the USGS. The furnished turbidity data were reviewed and approved by the USGS and published in the National Water Information System^52^.

### Propagation of a flood wave and sediment pulse

Streamflow and turbidity observations collected at the Seiad, Orleans, and Klamath monitoring stations (Fig. 1 and Table 1) were used to interpret the longitudinal propagation of a flood wave and sediment pulse composed of fire-scar material. In this context, “flood wave” refers to the rapid rise and fall of streamflow produced by surface runoff^55,56^, and the pulse of fire-scar material composed of ash, charcoal, sediment, and fire retardant is termed a “sediment pulse”. We investigated flood wave propagation using time series plots of streamflow (hydrographs) and sediment pulse propagation using time series plots of turbidity (turbidigraphs).

We calculated travel distances, longitudinal travel times, lag times, flood wave celerities (km/h), and sediment pulse velocities (km/h). Travel distances were computed from the McKinney Fire perimeter at the Humbug Creek confluence downstream to the Seiad, Orleans, and Klamath monitoring stations using published river distances^57^. Longitudinal travel times from the fire perimeter to each monitoring station for both the flood wave and sediment pulse were computed using the time differences between the end of the rain-on-wildfire event and the arrival of the flood wave and sediment pulse. Arrival times for the flood wave were determined using the date and time of streamflow peaks, while arrival times for the sediment pulse were determined using the date and time of turbidity peaks. We identified these dates and times using the maximum streamflow and turbidity values. Lag times between the arrival of the flood wave and sediment pulse were computed as the time difference between the arrival of the streamflow and turbidity peaks. Flood wave celerities and sediment pulse velocities were computed as river distance (km) divided by travel time (h), with travel times estimated as the difference between the end of the rainfall event and the arrival of the streamflow and turbidity peaks respectively.

### Water-quality impairments

We evaluated water-quality responses at the Seiad, Orleans, and Klamath stations using observations from July 28, 2022, to August 14, 2022. For each station, we assessed the streamflow response to monsoonal rainfall and analyzed the timing, magnitude, and duration of water-quality responses to the first flush of fire-scar material. Subsequently, we examined the timing, magnitude, and duration of the flood wave and sediment pulse, as well as the response of conductivity, dissolved oxygen, pH, and water temperature, to explore complex interactions between the flood wave, sediment pulse, and water-quality parameters.

To identify anomalous excursions in water quality indicative of wildfire effects, we focused on the Seiad station (Fig. 1) and compared the 2022 water-quality observations from July to September with historical data spanning 2012 to 2021. To assess the water-quality anomalies, we compared the timing of the maximum instantaneous (15 min) values in turbidity and conductivity to the minimum instantaneous (15 min) values in dissolved oxygen, pH, and water temperature. The historical turbidity data covers four years (2018–2021), while streamflow and other water-quality data span ten years (2012–2021).

## Results

### Rain-on-wildfire summary

**Fig. 2:**
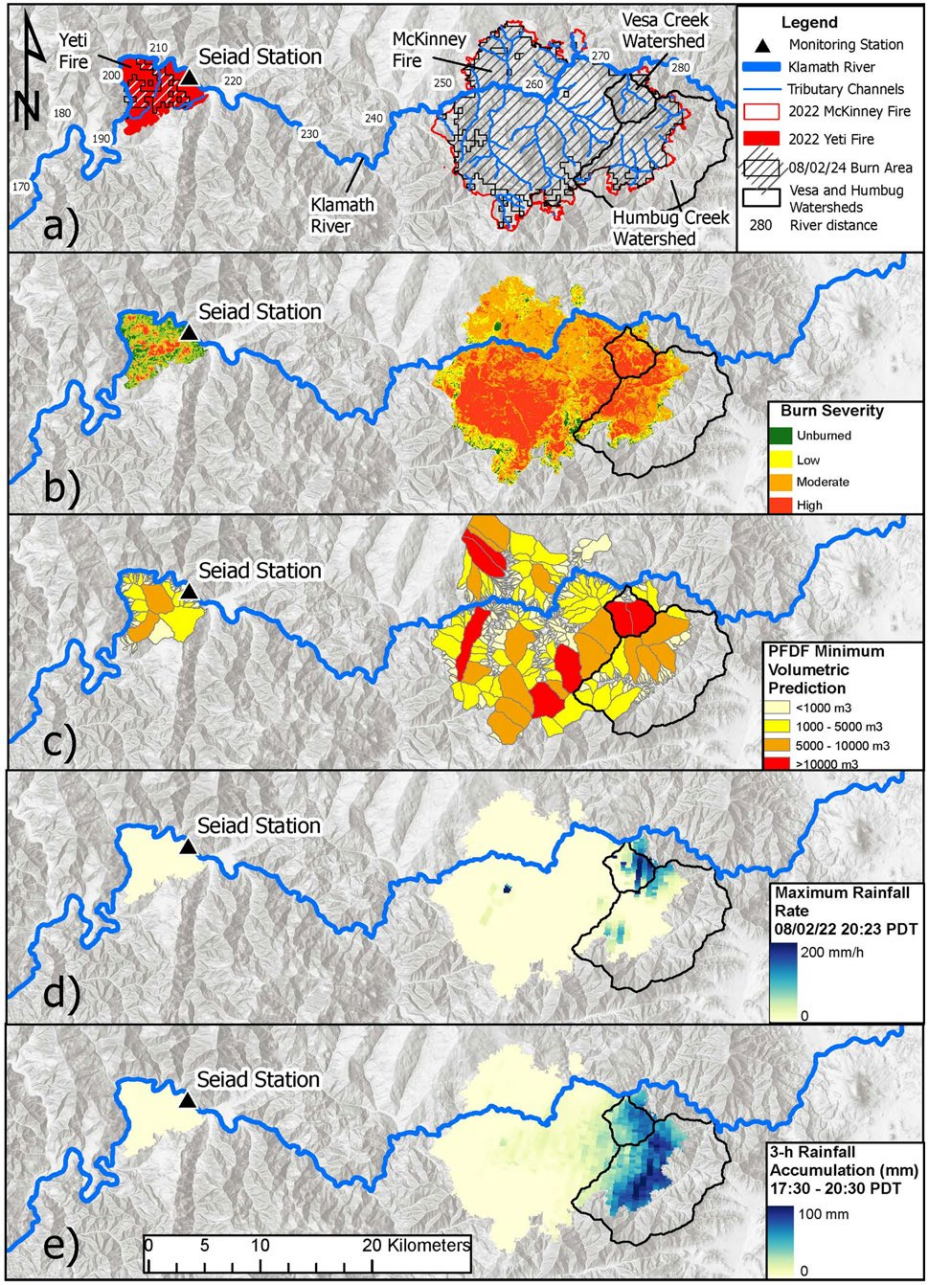
Geospatial datasets used to assess the downstream water-quality impacts of the 2022 McKinney rain-on-wildfire event on the main-stem Klamath River, California. The rain-on-wildfire event triggered sediment-laden flooding and post-fire debris flows (PFDFs) in the Vesa and Humbug watersheds, delivering the first flush of fire-scar material to the downstream receiving waters of the Klamath River. Panel (**a**) shows burned areas for the 2022 McKinney Fire and 2022 Yeti Fire on August 2, 2024, the location of the Seiad monitoring station, and river distances upstream from the Pacific Ocean. Thin blue lines represent tributary channels impacted by the Yeti Fire and McKinney Fire. Red outlines represent wildfire boundaries. Black outlines represent the Vesa and Humbug watersheds that produced sediment-laden flooding and PFDFs. Panel (**b**) shows burn severity maps^43^. Panel (**c**) shows estimates of the minimum predicted PFDF volumes computed using a design storm with a 15 min rainfall intensity of 40 mm/h^52^. Panel (**d**) shows maximum rainfall rates on August 2, 2024, at 20:23 PDT computed using NEXRAD 5-6 min rainfall rate estimates measured by the KMAX weather station (*NCDC, 2023a*). Panel (**e**) shows 3-h rainfall accumulations on August 2, 2024, between 17:30 and 20:30 PDT, computed from hourly accumulation estimates measured by the KMAX Station (*NCDC, 2023a*). The highest 3-h rainfall accumulations occurred in Humbug Creek, while the highest rainfall rates, burn severity, and erosion by PFDFs occurred in Vesa Creek. Figure created using ArcGIS Pro (v. 10.6; Esri, Redlands, CA, USA).

The Yeti Fire started on July 28, 2022, burned 32 km^2^, and was fully contained on September 1, 2022. The McKinney Fire began on July 29, 2022, burned 243 km^2^, and was fully contained on October 1, 2022. The Yeti Fire directly impacted 16 km of the main-stem river (river kilometers 198 to 214), while the McKinney Fire directly impacted 25 km (river kilometers 247 to 272). According to the MODIS-MCD64A1 Burned Area product analysis, 1% of the McKinney Fire burned from July 29–30, 2022, 89% burned from July 31 to August 2, 2022, and 10% burned on or after August 3, 2022 (Fig. 2a). The severity maps (Fig. 2b;^43^) indicate that the areas burned by the Yeti Fire were primarily unburned (22%) or low burn (40%) severity, while the areas burned by the McKinney Fire exhibited a predominance of moderate (46%) to high (37%) burn severity.

On August 2, 2022, an unsettled weather pattern initiated monsoonal flow from the south, directing warm, moist air into northern California. At approximately 17:30 PDT, monsoonal moisture produced a convective thunderstorm over the active perimeter of the McKinney Fire. The storm generated localized cells of intense rainfall lasting ~ 3 h from 17:30 to 20:30 PDT. Initially, the storm produced intense rainfall over the Humbug Creek watershed, triggering sediment-laden flooding. As the storm migrated into the steeper Vesa Creek watershed, sustained rainfall intensities triggered rill and gully erosion and PFDFs^58^.

In the Vesa and Humbug watersheds, estimated rainfall rates exceeded 200 mm/h (Fig. 2d), with 3 h rainfall accumulations surpassing 100 mm (Fig. 2e). In isolated cells, rainfall rates exceeded 150 mm/h for more than 30 min and 200 mm/h for over 5 min. Such extreme rainfall is uncommon during the summer low-flow season in the Klamath River basin. The recurrence interval for a rainfall event with a peak 15 min intensity of 100 mm/h in the Vesa and Humbug watersheds is over 1000 years, with an annual exceedance probability of less than 0.001%^46^.

During the rain-on-wildfire event, NEXRAD gridded precipitation maps show no rainfall over the Yeti Fire scar (Fig. 2d,e). The rainfall analysis indicated that localized high-intensity precipitation was confined to the the Vesa and Humbug watersheds within the active perimeter of the McKinney Fire. Although the Yeti Fire scar may have contributed runoff and sediment during other events, we assumed that any runoff and sediment mobilized and delivered from the low-severity Yeti Fire scar was minor during the rain-on-wildfire event.

Vesa Creek and Humbug Creek (Fig. 2) were the primary sources of sediment-laden flooding and runoff-initiated debris flows that delivered fire-scar material to the main-stem Klamath River. These two headwater basins are steep mountain watersheds with highly erodible hillslopes underlain by deeply weathered soils and regolith^58^. When monsoonal moisture moved from the headwaters of Humbug Creek into Vesa Creek (Fig. 2), orographic enhancement^59^ produced extreme rainfall intensities.

Post-event field reconnaissance indicated extensive rill and gully erosion of burned hillslopes in the Vesa and Humbug watersheds^58^. Intense rainfall exceeded the infiltration capacity of fire-affected soils and regolith, producing runoff that triggered rill and gully erosion, and creating sediment slurries that coalesced and transitioned into debris flows in areas underlain by deeply weathered regolith and colluvium. Runoff-initiated debris flows scoured channel reaches, uprooted riparian vegetation, and deposited debris fans in lower gradient reaches of Vesa Creek and Humbug Creek, where the tributary channels flow into the main-stem river.

NEXRAD estimates of rainfall rates over the Vesa and Humbug watersheds (Fig. 2d) were well above the maximum design storm thresholds computed for the McKinney PFDF hazard models. The minimum PFDF volumetric predictions for the McKinney Fire, computed using a maximum-intensity design storm with a 15 min rainfall rate of 40 mm/h (Fig. 2c;^52^, were 24,000 m^3^ for Vesa Creek and 57,000 m^3^ for Humbug Creek. The maximum PFDF volumetric predictions, computed using the same design storm, were 1,554,000 m^3^ for Vesa Creek and 3,676,000 m^3^ for Humbug Creek^52^. Summing these PFDF volumetric predictions suggests the potential sediment delivery to the main-stem Klamath River by PFDFs during the rain-on-wildfire event was likely more than 81,000 m^3^ and may have exceeded 5,230,000 m^3^.

### Propagation of a flood wave and sediment pulse

Studies that investigate the complex interactions between flood waves and sediment pulses during and following wildfires are uncommon because high-frequency monitoring in fire-prone areas is rare. To address this knowledge gap, we conducted a detailed analysis of the longitudinal propagation of the flood wave, sediment pulse, and water-quality impairments.

**Fig. 3:**
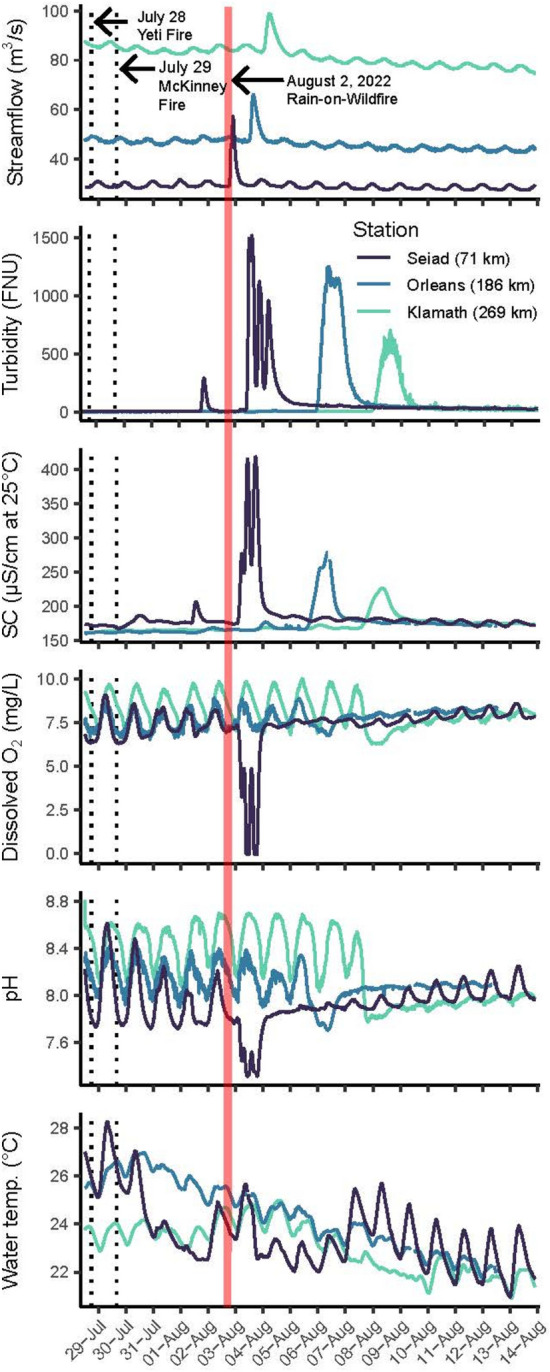
Continuous 15 min streamflow and water-quality observations showing the longitudinal propagation of a flood wave, sediment pulse, and associated water-quality impairments captured at three downstream monitoring stations^52–54^ following the 2022 McKinney rain-on-wildfire event along the main-stem Klamath River, California. Time-series graphs show peaks in streamflow that preceded the arrival of turbidity pulses. Spikes in turbidity and specific conductance (SC), and sags in dissolved oxygen (O_2_) and pH were in sync with the arrival of the turbidity pulse at each station. The duration of the rain-on-wildfire event is shown in each panel by the shaded red area. Water-quality impairments were most pronounced upstream, closest to the sediment pulse source areas, and generally decreased with increasing distance downstream. Monitoring station locations are reported in parentheses as kilometers (km) downstream from Humbug Creek, a primary sediment source watershed. Station descriptions are shown in (Table 1), and station locations are shown in (Fig. 1).

The rain-on-wildfire event created a flood wave and sediment pulse that propagated 71 km (Table 1) downstream from the confluence of Humbug Creek with the main stem (river kilometer 282), past the confluence with Vesa Creek (river kilometer 271), to the Seiad station (river kilometer 211). At 01:45 PDT on August 3, 2022, streamflow at the Seiad station began to rise, and at 05:45 PDT, a streamflow peak arrived approximately 9.25 h after the rainfall event ended (Table 2). Streamflow doubled from 29 to 58 m^3^/s over 4 h when the flood wave arrived (Fig. 3). The first turbidity peak occurred at 14:45 PDT (~ 9 h after the streamflow peak), a second peak arrived at 21:45 PDT (~ 16 h after the streamflow peak), and a third peak arrived on August 4 at 05:00 PDT (~ 23.25 h after the streamflow peak).

**Table 2:** Description of streamflow and turbidity peaks, travel times for a flood wave and sediment pulse, flood wave celerity, sediment pulse velocity, and lag time between the streamflow and turbidity peaks for three monitoring stations used to investigate patterns in the longitudinal propagation of water-quality impairments along the main-stem Klamath River, California.

USGS station	Date and time of streamflow peak (m/d/yyyy, hh:mm)	Flood wave travel time (h)	Flood wave celerity (km/h)	Date and time of turbidity peak (m/d/yyyy, hh:mm)	Sediment pulse travel time (h)	Sediment pulse velocity (km/h)	Lag time (h)
Klamath River at Seiad Valley	8/3/2022 05:45	9.25	6.6	8/3/2022 14:45	18.25	3.30	9.00
Klamath River at Orleans	8/3/2022 23:45	27.25	4.2	8/6/2022 09:15	84.75	1.4	15.50
Klamath River near Klamath	8/4/2022 13:45	41.25	2.0	8/8/2022 10:15	133.75	0.6	23.25

As the flood wave and sediment pulse propagated downstream past the Yeti Fire perimeter (river kilometer 214 to 198) to the Orleans station (river kilometer 96) and Klamath station (river kilometer 13), the turbidity peaks coalesced into a single peak, and the magnitude of the streamflow peaks and water-quality impairments diminished (Fig. 3). At the Orleans station, the streamflow peak arrived 27 h after the rain event ended, and a single turbidity peak arrived 57.5 h after the streamflow peak (Table 2). Further downstream at the Klamath station, the streamflow peak arrived 41 h after the rain event ended, and a single turbidity peak arrived 92.5 h after the streamflow peak (Table 2).

The rain-on-wildfire event occurred during the low-flow season, with minimal additions of streamflow and sediment between the monitoring stations, allowing us to compute flood wave celerities and sediment pulse velocities. Herein, flood wave celerity is defined as the speed of a flood wavefront as it propagates through a river channel. We assumed that floodwaters and sediment initially exited the Vesa and Humbug watersheds immediately after the monsoonal rainfall event on August 2 at 20:30 PDT. If floodwaters and fire-scar material entered the main-stem Klamath River earlier, the computed travel times would be underestimated, leading to an overestimation of the associated flood wave celerity and sediment pulse velocity.

The flood wave and sediment pulse became decoupled as they propagated downstream, and the estimated lag times progressively increased with distance (Table 2). Consequently, the flood wave traveled 2 to 3.3 times faster and outpaced the sediment pulse. Between the Humbug Creek confluence and the Seiad station, the flood wave’s celerity was 6.6 km/h, while the velocity of the first sediment pulse to arrive was 3.3 km/h. From the Seiad station to the Orleans station, the flood wave celerity declined to 4.2 km/h, while the velocity of the coalesced sediment pulse decreased to 1.4 km/h. Between the Orleans station and the Klamath station, the flood wave celerity further declined to 2.0 km/h, and the sediment pulse velocity dropped to 0.6 km/h.

### Water-quality impairments and fish kill

During the rain-on-wildfire event, we observed excursions in water quality at the Seiad, Orleans, and Klamath stations, including spikes in turbidity and conductivity, as well as sags in dissolved oxygen and pH (Fig. 3). Herein, “sag” is defined as a rapid decline in a water-quality parameter followed by recovery plotted over time. At all three monitoring stations, turbidity and conductivity spiked as dissolved oxygen and pH declined (Fig. 3). In contrast, the response of water temperature was out of sync with other water-quality responses at all three monitoring stations, indicating that air temperature or other factors influenced changes in water temperature.

**Fig. 4:**
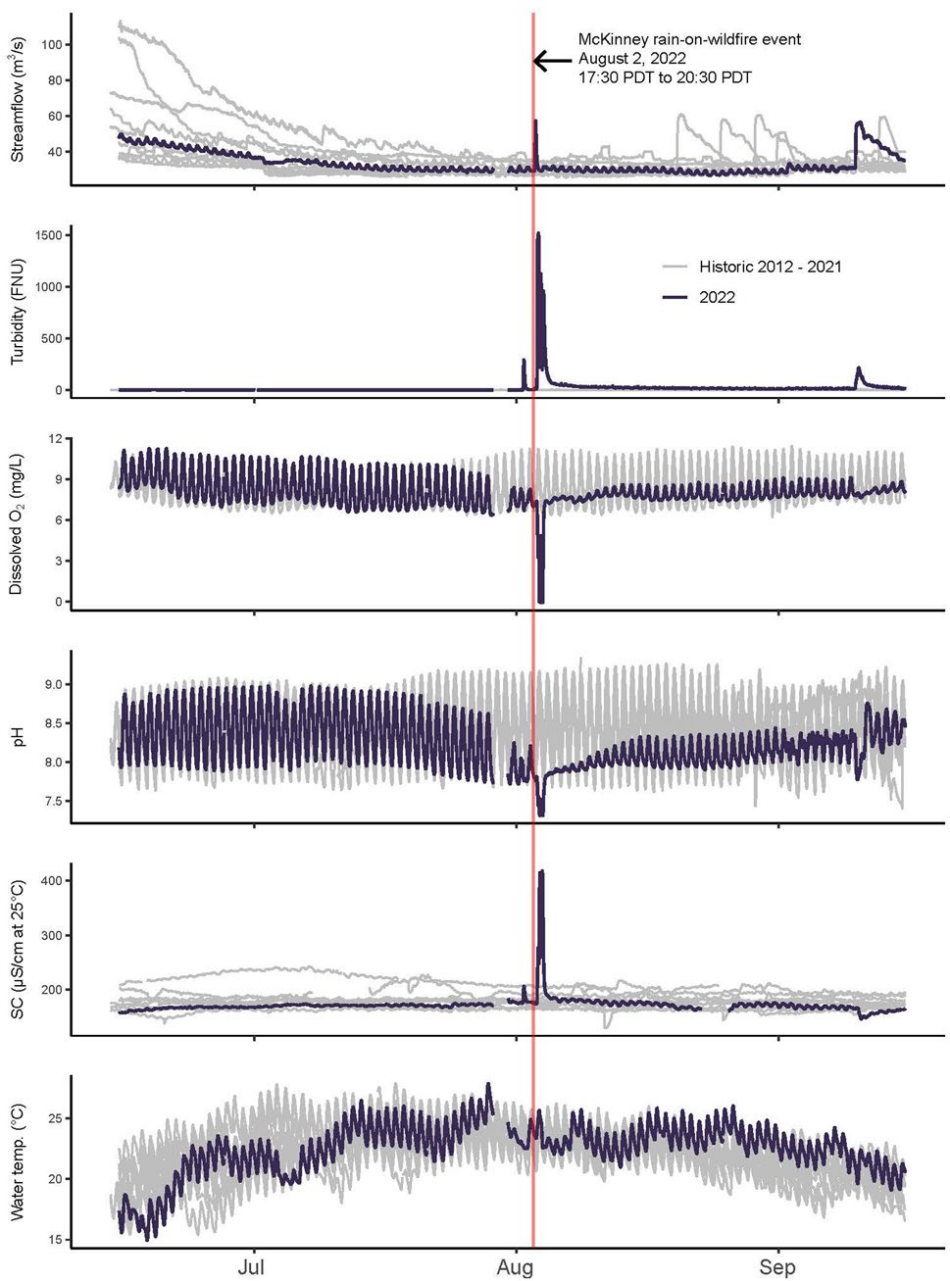
Continuous 15 min streamflow and water-quality observations showing unprecedented and acute water-quality impairments captured at the Seiad station, within a 95 km fish kill zone and located 211 km upstream from the Pacific Ocean and 71 km downstream from the Humbug Creek confluence along the main-stem Klamath River, California. Time-series graphs show streamflow, turbidity, specific conductance (SC), dissolved oxygen (O_2_), pH, and water temperature observations from July to September and show the 2022 data collected before, during, and after the McKinney rain-on-wildfire event in the context of historical data spanning 2012 to 2021, with turbidity data spanning 2018 to 2021. The Seiad station description is in (Table 1), the Seiad station location is shown in (Figs. 1, 2), and the fish kill zone is shown in (Fig. 1).

At the Seiad station, located within the 95 km fish kill zone (Fig. 1), we observed three distinct spikes in turbidity and conductivity, as well as three separate sags in dissolved oxygen and pH (Fig. 3), which were unprecedented in historical observations (Fig. 4). Turbidity increased and exceeded 1,000 FNU for over 5 h, and the actual turbidity values may have been higher than the recorded values because the accuracy of the turbidity sensors used in this study declined above 1,000 FNU^51^. Specific conductance increased from 175 to 415 µS/cm (at 25 °C). Anoxia occurred when dissolved oxygen dropped to zero for a total of 5.25 h and was likely the primary cause of the fish kill. Dissolved oxygen remained below 5 mg/L, and pH remained below 7.5 for over 16 h. Daytime water temperatures briefly increased by 0.5 °C on August 3, 2022, then cooled by 1.0 °C from August 4 through August 7, 2022.

As the sediment pulse propagated further downstream, the magnitude of water-quality impairments attenuated (Fig. 3). At the Orleans station, the arrival of the sediment pulse coincided with a spike in turbidity that exceeded 1,000 FNU for more than 4 h, while conductivity increased from 168 to 278 µS/cm (at 25 °C), dissolved oxygen decreased by 0.7 mg/L from 7.4 to 6.7 mg/L, and pH decreased by 0.4 units from 8.1 to 7.7. At the Klamath station, the arrival of the sediment pulse coincided with elevated turbidity levels that remained above 550 FNU for over 8 h, conductivity increase from 168 to 278 (µS/cm at 25 °C), dissolved oxygen declined by 1.1 mg/L from 7.4 to 6.3 mg/L, and pH decreased by 0.3 units from 8.1 to 7.8.

A comparison of the 2022 water-quality observations, collected from July through September at the Seiad station, with historical observations from 2012 to 2021 shows excursions indicative of wildfire effects (Fig. 4). Thresholds for interpreting excursions were turbidity above 80 FNU, conductivity above 250 µS/cm (at 25 °C), pH below 7.5, and dissolved oxygen below 6 mg/L. The historical data showed no large excursions. In stark contrast to the historical data, the rain-on-wildfire event created unprecedented spikes in turbidity that exceeded 1,000 FNU and conductivity that exceeded 400 µS/cm (at 25 °C), pH sags to 7.3,  and periods of hypoxia (dissolved oxygen < 2 mg/L) and anoxia (dissolved oxygen = 0 mg/L; Fig. 4).

## Discussion

In rivers, streamflow and turbidity peaks rarely occur simultaneously^56^, and counterclockwise hysteresis following wildfires is common^60^. Counterclockwise hysteresis occurs when turbidity is lower on the rising limb than on the falling limb of a hydrograph and may reflect distal sediment sources with longer travel times or slower mobilization^61,62^. Conversely, clockwise hysteresis occurs when turbidity is higher on the rising limb than on the falling limb of a hydrograph, which may indicate sediment contributions from proximal sources with shorter travel times or rapid mobilization. During the rain-on-wildfire event, lag times between the arrival of the flood wave and the sediment pulse progressively increased as both propagated downstream, causing strong counterclockwise hysteresis.

Unless additional sediment is entrained from a tributary or main-stem source, hysteresis will progressively increase with downstream distances^55,56^, due to differences in flood wave celerity and sediment pulse velocity^63,64^. Celerity and velocity often differ because they are influenced by distinct processes^65^. Generally, celerity is defined as the rate at which a disturbance in flow propagates through a flow domain, whereas velocity is the rate at which a distinct mass changes its position, characterized by both speed and direction.

The longitudinal propagation of a flood wave through a river channel is controlled by wave celerity, whereas sediment pulse velocities are controlled by the mean streamflow velocity^66,67^. During the rain-on-wildfire event, flood wave celerity controlled the hydrograph and the arrival time of the streamflow peak, while mean streamflow velocity controlled the transport of suspendable-sized fire-scar material and the arrival time of the turbidity peak. Generally, flood wave celerities are considerably faster than sediment pulse velocities^67^, and the progressive lag of a sediment pulse behind a flood wave is most apparent when a single storm event produces a fine-sediment pulse that travels long distances downstream^55,56^. The rain-on-wildfire event illustrated these principles. When the flood wave outpaced the sediment pulse, traveling downstream 2 to 3.3 times faster, the flood wave became decoupled from the sediment pulse, the dilution effect of the floodwaters decreased, and downstream water-quality impairments intensified.

During the rain-on-wildfire event, water-quality impairments varied longitudinally between monitoring stations. This variability may be linked to differences in river processes, land use, and geology, or to additions of streamflow, reactive carbon, or reactive metals. While ash inputs from wildfire runoff can increase pH due to the alkaline nature of ash material^6,11^, decreases in pH (~ 0.75 units) can be linked to biological or chemical processes during periods of very high turbidity^9,18^. Dissolved oxygen sags to zero (anoxia) observed at the Seiad station may suggest rapid microbial respiration, which could reduce pH due to the production of carbon dioxide^68^ or may indicate rapid oxidation of reduced metals releasing hydrogen ions into solution, leading to a decrease in pH^69^. The spikes in conductivity (a measure of dissolved ions) may be attributed to the influx of ash and mineralized soils from areas with high burn severity^9^.

This study used streamflow and water-quality observations collected at existing stations along the main-stem Klamath River to investigate water-quality impairments. The number and location of the pre-existing stations effectively captured longitudinal variations in streamflow and water quality with sufficient accuracy and detail to support analyses of the longitudinal propagation of water-quality impairments. However, the magnitude and possibly the duration of these impairments were likely greater within the McKinney Fire perimeter and immediately downstream of the Humbug Creek and Vesa Creek confluences.

The magnitude of water-quality responses diminished as the flood wave and sediment pulse propagated from the Seiad station downstream to the Orleans and Klamath stations. Clearwater additions from tributaries serve as a primary mechanism for mitigating extreme water-quality impacts in wildfire-affected rivers. On August 1, 2022, the day before the rain-on-wildfire event, mean daily streamflow was 29 m^3^/s at the Seiad station, 47 m^3^/s at the Orleans station, and 84 m^3^/s at the Klamath station. Muted water-quality responses at the Orleans and Klamath stations may be attributed to dilution by the addition of tributary streamflow or higher decomposition rates closer to sources of the fire-scar material, resulting in less reactive material being transported downstream.

In rivers, the consumption of dissolved oxygen occurs through two mechanisms: biochemical oxygen demand (BOD) and chemical oxygen demand (COD). The impacts of BOD and COD on dissolved oxygen during and following large wildfires is an emerging area of research^9,10,18^. In this study, the data required to differentiate between rates of BOD and COD was unavailable. Either BOD, COD, or a combination of both may have contributed to the observed responses in dissolved oxygen at the monitoring stations.

During and following large wildfires, reaeration is a primary mechanism for buffering declines in dissolved oxygen caused by BOD and COD^70^. Variations in turbulence and reaeration along the main-stem Klamath River may have influenced dissolved oxygen responses. The factors influencing turbulence and reaeration along the main-stem of the Klamath River include channel gradient, water depth, stream flow velocity, wind speed, wave action, and roughness^27^.

Higher-gradient reaches have more mixing energy available to facilitate faster reaeration, and turbulent river reaches are better able to buffer declines in dissolved oxygen during and following wildfires^70^. The Seiad and Orleans stations are in high reaeration reaches characterized by steeper channel gradients, larger bed material, higher relief riffles, and greater turbulence. In contrast, the Klamath station is in a low reaeration reach, characterized by gentler channel gradients, smaller bed material, longer pools, lower relief riffles, and less turbulence^27,34^. The Seiad station experienced the longest duration of elevated turbidity and dissolved oxygen sags to zero (anoxia). The magnitude and duration of dissolved oxygen sags at the Orleans and Klamath stations were similar. Although turbidity was substantially lower in the Klamath reach, the Orleans reach reaerated more quickly.

Following the rain-on-wildfire event, the recovery of diel variations in dissolved oxygen was delayed (Fig. 3). Generally, diel variation in dissolved oxygen increases with increasing gross primary production (GPP) and ecosystem respiration. GPP can be influenced by reaeration; in reaches with relatively stable reaeration, decreases in primary production can cause lower diel variations in dissolved oxygen. During and following the rain-on-wildfire event, dissolved oxygen responses suggest that GPP rates were suppressed. Suppressed GPP may have been caused by external factors, such as reduced light or declines in aquatic plant and algal biomass^71^.

The propagation of sediment pulses can scour and bury benthic organisms (epilithic algae, filamentous green algae, and rooted aquatic macrophytes) that contribute to GPP^72,73^. Moreover, extreme turbidity events can severely limit the light necessary for primary production in all but the shallowest locations^74,75^. Scour and burial of primary producers, along with extreme turbidity during the rain-on-wildfire event, likely contributed to GPP suppression.

At the Seiad station, turbidity declined following the initial spike and remained elevated through mid-September, while the diel variation in dissolved oxygen recovered but remained muted through mid-September (Fig. 4). Following the rain-on-wildfire event, dilution flows to mitigate fish disease during the fall salmon run were released from upstream reservoirs formerly impounded by dams recently removed in 2023 and 2024 (Fig. 1). At the Seiad Station, the dilution flows created a small turbidity spike and low amplitude sags in dissolved oxygen (Fig. 4) illustrating long-lasting impacts of the rain-on-wildfire event on GPP along 71 km of the main-stem Klamath River.

Water-quality impairments are stressful and can be lethal when fish are exposed to high suspended-sediment concentrations^76^ or dissolved oxygen concentrations below 2 mg/L^77^. The 2022 McKinney rain-on-wildfire event produced a flood wave, sediment pulse, and water-quality impairments that propagated 269 km downstream from the confluence of Humbug Creek to the Klamath station, producing a 95 km fish kill zone. Although there was no official census of the fish kill caused by the rain-on-wildfire event, field observations by the Karuk Tribe Fisheries Department indicated that approximately 10,000 fish died. The fish kill primarily included non-anadromous resident fish species, but out-migrating anadromous juveniles and incoming anadromous fall spawners may have also been affected.

The rain-on-wildfire event produced the first flush of fire-scar material during the summer low-flow season on the hottest day of the year, when base flows and clear water contributions from tributaries were at a minimum. Low flow conditions intensified downstream water-quality and ecological impairments, which were further exacerbated by hysteresis, as the flood wave outpaced the sediment pulse. During the summer months, salmon typically seek cold-water refugia. If this event had occurred during the fall spawning run, it could have had catastrophic effects on threatened salmon populations. Given that wildfires in the western US primarily occur during the low flow season, rain-on-wildfire events represent a unique class of first-flush events with a high likelihood of causing extreme water-quality and ecological impairments in downstream receiving waters.

To the best of our knowledge, only three published studies exist worldwide that document anoxia during or following large wildfires. These studies include the 2011 Las Conchas Fire^18^, the 2022 Hermit’s Peak-Calf Canyon Fire^12^, and this study of the 2022 McKinney rain-on-wildfire event. There are commonalities among these studies. All three fires occurred in forested basins, and high-intensity monsoonal rainfall triggered a first flush event, leading to severe water-quality impairments and anoxia in downstream river reaches. In each case, anoxia was likely driven by increases in BOD and COD stimulated by the influx of organic-rich, chemically reduced fire-scar material. The scarcity of studies documenting anoxia during or following wildfires may reflect the scarcity of high-frequency monitoring capable of capturing transient occurrences of anoxia in wildfire-affected rivers rather than the infrequency of anoxia events.

Predictions of a more fire-prone future across the western US^1^, accompanied by increases in probable maximum precipitation^78^, suggest that fire-related sediment delivery^79^ and downstream water-quality impairments^3^ will likely increase. Despite the Klamath River basin being in California’s wettest region, it is experiencing significant increases in the frequency, severity, and size of wildfires^80^.

In a more fire-prone future, low-intensity fires are less likely to cause downstream water-quality and ecological impairments^81,82^. In the absence of intense rainfall, naturally occurring and controlled small and low-intensity fires, such as the 2022 Yeti Fire, may help reduce the availability of dry fuels in the Klamath River basin without noticeably affecting downstream water quality and ecology.

Rain-on-wildfire events are rare but may become more frequent with increasingly extreme future climates. The 2022 McKinney rain-on-wildfire event was extraordinary, resulting in unprecedented downstream water-quality impairments. Fire-prone areas that experience seasonal drought and late summer monsoonal rainfall are particularly vulnerable to rain-on-wildfire events. Future climatic warming and the strengthening of warm-season monsoonal storms^83^ under drier future climates^84^ could amplify the frequency and severity of rain-on-wildfire events across the western US.

## Conclusions

The longitudinal propagation of water-quality and ecological responses to wildfires remains poorly understood due to the scarcity of high-frequency monitoring in fire-prone areas. The scarcity of studies documenting dissolved oxygen sags to zero (anoxia) may reflect a lack of monitoring data rather than the infrequency of wildfire-driven anoxia and related fish kills. Rain-on-wildfire events represent a unique class of first-flush events with a greater likelihood of causing extreme water-quality and ecological impairments in downstream receiving waters. This study highlights the elusive and transient nature of extreme water-quality and ecological impairments during active wildfires, providing novel insights into the potential role of rain-on-wildfire events in an increasingly fire-prone future. The findings emphasize the importance of high-frequency monitoring in fire-prone areas to assess transient water-quality impairments during active wildfires and to inform forest and river management strategies aimed at enhancing ecosystem resilience to wildfires.

## Data Availability

Data that support the findings of this study are publicly available in databases managed by the National Climate Data Center (NCDC^41^; https://www.ncdc.noaa.gov/nexradinv/), National Aeronautics and Space Administration (Giglio^39^; 10.5067/MODIS/MCD64A1.061), U.S. Geological Survey (USGS^52^; 10.5066/F7P55KJN), Karuk Tribe Water Quality Department (Karuk Tribe^53^, https://waterquality.karuk.us/), and Yurok Tribe Environmental Department (Yurok Tribe^54^, https://www.yuroktribe.org/environmental-department/). Please contact Karuk and Yurok tribal corresponding authors directly for data inquiries.
